# A Novel Five Gene Signature Derived from Stem-Like Side Population Cells Predicts Overall and Recurrence-Free Survival in NSCLC

**DOI:** 10.1371/journal.pone.0043589

**Published:** 2012-08-29

**Authors:** Deepak Perumal, Sandeep Singh, Sean J. Yoder, Gregory C. Bloom, Srikumar P. Chellappan

**Affiliations:** 1 Department of Tumor Biology, H. Lee Moffitt Cancer Center & Research Institute, Tampa, Florida, United States of America; 2 Molecular Genomics Core Facility, H. Lee Moffitt Cancer Center & Research Institute, Tampa, Florida, United States of America; 3 Department of Biomedical Informatics, H. Lee Moffitt Cancer Center & Research Institute, Tampa, Florida, United States of America; Roswell Park Cancer Institute, United States of America

## Abstract

Gene expression profiling has been used to characterize prognosis in various cancers. Earlier studies had shown that side population cells isolated from Non-Small Cell Lung Cancer (NSCLC) cell lines exhibit cancer stem cell properties. In this study we apply a systems biology approach to gene expression profiling data from cancer stem like cells isolated from lung cancer cell lines to identify novel gene signatures that could predict prognosis. Microarray data from side population (SP) and main population (MP) cells isolated from 4 NSCLC lines (A549, H1650, H460, H1975) were used to examine gene expression profiles associated with stem like properties. Differentially expressed genes that were over or under-expressed at least two fold commonly in all 4 cell lines were identified. We found 354 were upregulated and 126 were downregulated in SP cells compared to MP cells; of these, 89 up and 62 downregulated genes (average 2 fold changes) were used for Principle Component Analysis (PCA) and MetaCore™ pathway analysis. The pathway analysis demonstrated representation of 4 up regulated genes (*TOP2A, AURKB, BRRN1, CDK1*) in chromosome condensation pathway and 1 down regulated gene *FUS* in chromosomal translocation. Microarray data was validated using qRT-PCR on the 5 selected genes and all showed robust correlation between microarray and qRT-PCR. Further, we analyzed two independent gene expression datasets that included 360 lung adenocarcinoma patients from NCI Director's Challenge Set for overall survival and 63 samples from Sungkyunkwan University (SKKU) for recurrence free survival. Kaplan-Meier and log-rank test analysis predicted poor survival of patients in both data sets. Our results suggest that genes involved in chromosome condensation are likely related with stem-like properties and might predict survival in lung adenocarcinoma. Our findings highlight a gene signature for effective identification of lung adenocarcinoma patients with poor prognosis and designing more aggressive therapies for such patients.

## Introduction

Lung cancer remains the leading cause of cancer-related deaths worldwide [1]. Non-small cell lung cancer (NSCLC) accounts for 85% of all lung cancers and the average 5 year relative survival rate among NSCLC patients is only 15% [2]. The recurrence rate ranges from 35–50% among early stage non-small cell lung cancer patients. To date, there is no fully-validated and clinically applied prognostic gene signature for personalized treatment [3]. It remains a critical challenge to determine the risk for recurrence in early-stage cancer patients. Most important challenge in lung cancer studies is identifying patients at high risk for recurrence after surgical resection, as well as patients who would benefit from adjuvant treatment [4].

The emerging use of biomarkers enables to make treatment decisions based on the specific characteristics of individual patients and their tumor, instead merely on population statistics [5]. The prevalence of lung cancer as the primary cause of cancer death in the United States has led to renewed efforts to obtain biomarker signatures that provide either prognostic or predictive information to guide therapy for individual patients (i.e., “personalized medicine”) [6]. Multiple genome-wide expression studies have demonstrated the usefulness of this approach for lung cancer prognosis [7].

Gene-expression profiling by means of microarrays and reverse-transcriptase polymerase chain reaction (RT-PCR) is useful for classifying tumors and predicting prognosis for patients with various types of cancer, including lung cancer [8], [9], [10]. However the use of microarrays in clinical practice is limited by the large number of genes used in gene profiling and lack of both reproducibility and independent validation [11], [12], [13]. Although microarray has been successfully used to predict clinical outcomes and survival, gene-expression profiles can vary according to the microarray platform and the analytic strategy used [14], [15].

Cancer cells with stem cell like properties in particular have been proposed to play a critical role in metastatic progression and resistance to commonly used chemotherapeutic agents [16]. These cells can be identified by various functional assays and using specific cell-surface markers. While cell surface markers have been used to identify stem like cells in various cancers, such markers have been difficult to identify in non-small cell lung cancers. In addition to cell surface markers, stem like cells have been isolated by their ability to efflux Hoechst 33342 dye and are referred to as the “side population” (SP) cells [17]. Side population cells have been shown to be enriched for tumor-initiating [17] and chemotherapy-resistant cells [18]. Flow cytometric analysis can be used to isolate SP or non-SP cells (Main population cells MP), which are more differentiated and has low tumor initiating properties.- Recent studies indicate that SP is an enriched source of lung tumor–initiating cells with stem cell like properties and may be an important target for effective lung cancer therapy [19].

In this study, we performed a gene expression analysis to assess whether gene expression profiles of side population and main population cells might have clinical relevance in predicting prognosis. Identification of gene signatures for outcomes can be expected to improve the clinical management of non-small cell lung cancer, since patients predicted to have poor prognosis can be subjected to more aggressive therapeutic strategies or closer surveillance. To achieve this, a list of genes whose expression was statistically different in SP and MP cells was generated and their clinical relevance tested on publicly available lung adenocarcinoma microarray data from the s NCI Director's Challenge set [20] and Sungkyunkwan University (SKKU) dataset [21]. A functional pathway analysis then revealed that the signature genes had interactions with well-established chromosome condensation pathways, indicating potential roles of the signature genes incancer. Studies have shown that multiple biochemical steps in chromosome condensation pathways are altered in cancer. These include modifications of histones and aberrations in Holliday junctions [22], [23]. Mitotic cell death can also occur as a result of premature chromosome condensation [24], [25]. Hence we hypothesize that since defects in chromosome condensation are correlated with cancers, it is probable that our signature genes might be contributing to oncogenesis. Quantitative RT-PCR analyses on the isolated SP and MP cells confirmed the gene expression patterns observed in the microarray data. In this study we show a five-gene signature that is closely associated with survival of patients with NSCLC. Further, the five-gene signature is an independent predictor of relapse-free and overall survival.

## Results

### Microarray analysis

**Figure 1:**
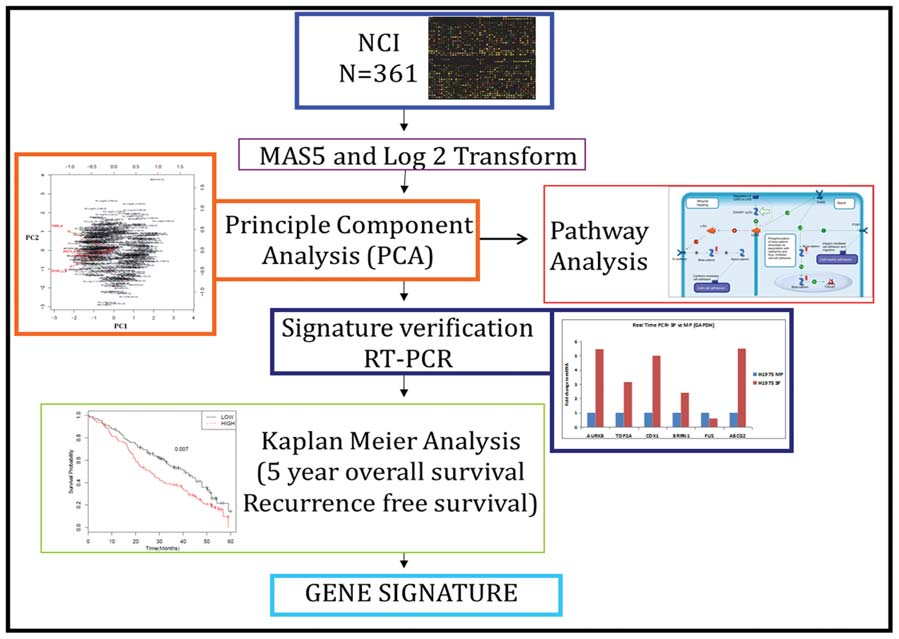
Flow chart showing the methodology followed for identifying gene signatures. Microarray was performed on 4 lung cancer cell lines A549, H1650, H460 and H1975. Total RNA extracted from SP and MP samples were used to generate cRNA targets, which were subsequently hybridized to Human Genome U133A plus 2.0 oligonucleotide arrays. Raw data was processed by log_2_ transformation of the expression values, and the mean center expression level for each gene was determined. Further obtained genes that were over or under-expressed in all 4 cell lines, whose expression was at altered least two fold. Further pathway analysis was carried out using MetaCore™ pathway database.

**Figure 2:**
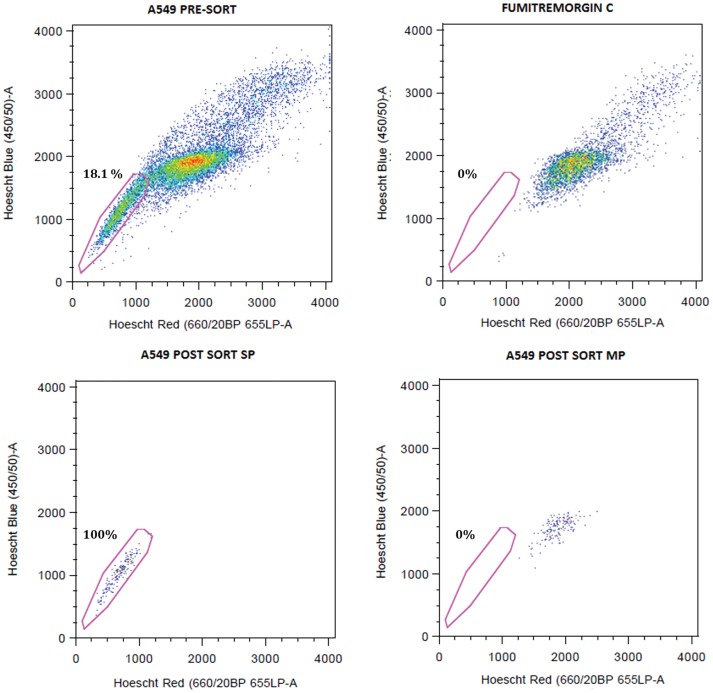
Sorting of SP and MP cells in A549 cells by FACS analysis. Appearance of SP cells as a tail emerging from main population is shown in (A). Inhibition of ABCG2 activity by Fumitremorgin C displayed complete loss of SP phenotype and confirmed the SP identity as well as gating strategy (B). Panel (C) shows sorted SP cells and panel (D) shows MP cells.

**Figure 3:**
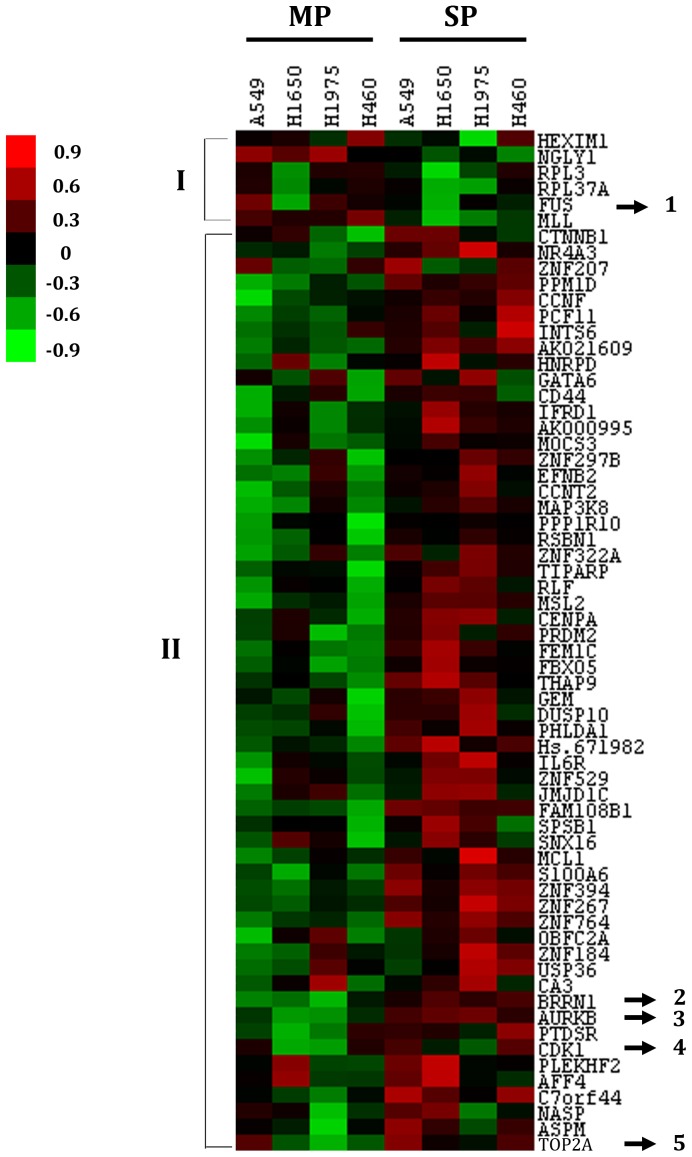
Heat map showing the expression pattern of 64 genes in lung adenocarcinoma. Differentially expressed genes in SP vs. MP cells in 4 NSCLC cell lines. A total of 64 genes (58 upregulated and 6 downregulated) with expression levels that showed at least two fold difference across 4 cell lines were selected for hierarchical clustering analysis. Two clusters shown here represent downregulated (I) and upregulated genes (II). The 5 genes that predicted significant prognosis are marked in the map by numbers. The color in red or green reflects relative high or low expression levels, respectively as indicated in the scale bar (log_2_ transformed scale).

**Figure 4:**
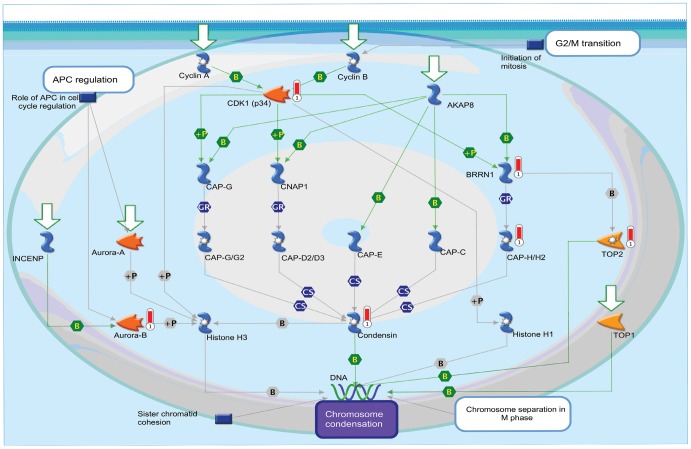
Chromosome Condensation Pathway. The pathway shows the role of 4 genes *AURKB*, *TOP2A*, *CDK1*, *BRRN1* (also known as NCAPH) in chromosome condensation a ubiquitous process in most eukaryotic cells.

A flowchart showing the different steps followed in finding the gene signature is shown in Figure 1. Four lung cancer cell lines A549, H1650, H460 and H1975 were subjected to FACS (Fluorescence activated cell sorting) analysis for sorting SP and MP cells. A representative sorting for A549 cells is shown in Figure 2A; inclusion of fumitremorgin C abolishes the SP (Figure 2B); this allowed setting the gate for sorting only SP cells accurately. Figures 2C and 2D show the sorted SP and MP cells respectively. The mRNA expression profiles were measured using Affymetrix Expression Console™ software and the data were analyzed. We found 354 up and 126 down-regulated genes common in all 4 cells lines (summarized in Table 1). Further we selected 89 upregulated (average 2 fold changes) and 62 downregulated (average 2 fold changes) genes out of which only 64 genes (58 upregulated and 6 downregulated) matched with the NCI Directors challenge set Affymetrix platform 133A. The heat map shows the expression levels of these genes in MP and SP for the 4 cell lines. The heat map represents two distinct clusters, cluster I representing the 6 downregulated genes and cluster II representing 58 upregulated genes (Figure 3). Further, the data from the microarray experiment was also used to assess if there were any significant pathways associated with those genes. For this analysis, the 89 upregulated (average 2 fold changes) and 62 downregulated (average 2 fold changes) were used for Metacore™ pathway analysis and this resulted in top 10 pathways with significant *p* values (Table 2). Results from the analysis showed that the signature genes interact with major pathways. The top pathway represented by chromosome condensation pathway showed 4 upregulated genes in (Table 3, Figure 4) and 1 down regulated gene FUS represented in chromosomal translocation pathway. The 5 significant genes obtained from pathway analysis were *TOP2A*, *AURKB*, *BRRN1*, *CDK1* and *FUS*.

**Table 1:** Microarray analysis data from 4 cell lines (A549, H1650, H460 and H1975).

MICROARRAY DATA SET	UP REGULATED	DOWN REGULATED
MUTANT EGFR CELL LINES H1650, H1975	1083	673
MUTANT K-RAS CELL LINES A549, H460	1128	314
ALL 4 CELL LINES	354	126
AVERAGE 2 FOLD CHANGE	89	62

The data analyzed resulted in 354 up-regulated and 126 down-regulated genes in (SP) common in all 4 cells lines. An average fold change of 2 was used for Principle Component analysis (PCA) and for identifying gene signatures.

**Table 2:** Top 10 significant pathways for the differentially regulated genes.

	Pathway Maps	pValue	Ratio	
1	Cell cycle: Chromosome condensation in prometaphase	1.2E-09	5	21
2	Cell cycle: Transition and termination of DNA replication	5.841E-07	4	28
3	Cell cycle: Role of APC in cell cycle regulation	0.000001	4	32
4	Cell cycle: Role of SCF complex in cell cycle regulation	0.002	2	29
5	Cytoskeleton remodeling: Reverse signaling by ephrin B	0.002	2	31
6	Cell cycle: Spindle assembly and chromosome separation	0.003	2	33
7	Cell cycle: The metaphase checkpoint	0.003	2	36
8	Apoptosis and survival: BAD phosphorylation	0.004	2	42
9	Development: WNT signaling pathway	0.007	2	53
10	Cell adhesion: Role of CDK5 in cell adhesion	0.022	1	9

For this analysis, the 89 up regulated (average 2 fold changes) and 62 down regulated genes (average 2 fold changes) were used for Metacore™ pathway analysis and this resulted in top 10 pathways with significant *p* values.

**Table 3:** List of five gene signatures from the microarray data.

	Gene Symbol	Gene Description
1	TOP2A	DNA topoisomerase II, alpha
2	AURKB	Aurora Kinase B
3	CDK1	Cyclin-Dependent Kinase 1
4	BRRN1	Non-SMC condensin I complex, subunit H
5	FUS	Fused in Sarcoma

The microarray data was examined to assess whether there are any significant pathways associated with those genes. Results from the analysis showed that the signature genes interact with major pathways. The top pathway represented by chromosome condensation pathway showed 4 up regulated genes in chromosome condensation pathway and 1 down regulated gene *FUS* represented in chromosomal translocation pathway.

### Real Time PCR validation

**Figure 5:**
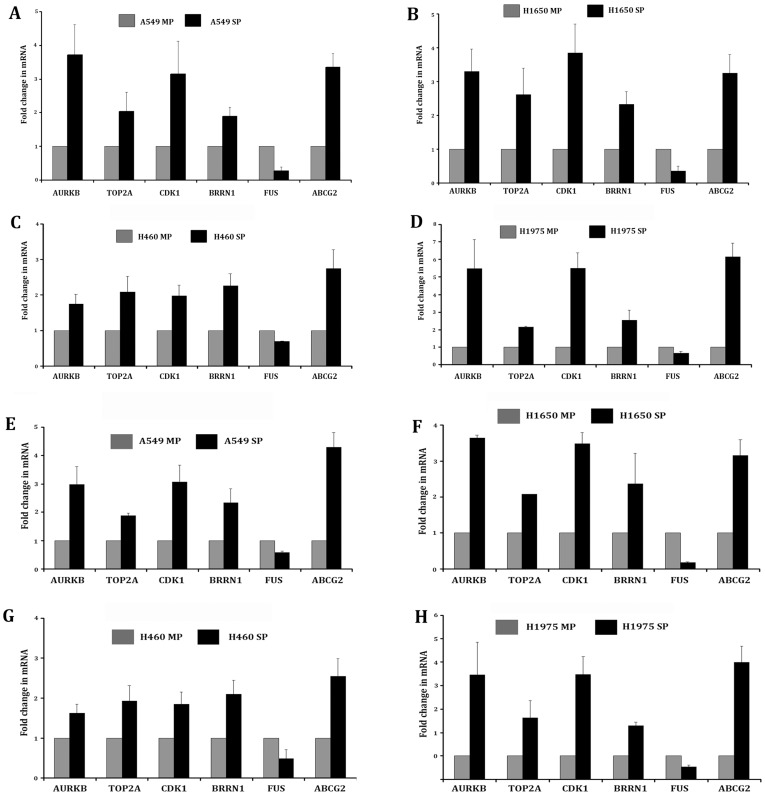
Validation of the microarray data by Quantitative Real Time PCR for the 5 genes in 4 NSCLC cell lines. RNA was extracted from MP and SP cells for A549 (A), H1650 (B), H460(C) and H1975 (D) cell lines. qRT-PCR was performed with the cDNA of the 4 cell lines with 18S as internal control and the up regulation of the 4 genes *TOP2A*, *AURKB*, *BRRN1* and *CDK1* in SP and down regulation of *FUS* in the side-population of all the cell lines was observed. *ABCG2* was used as a positive control for a gene overexpressed in SP cells. Similar experiments were conducted, using *GAPDH* as an internal control on A549 (E), H1650 (F), H460 (G) and H1975 (H) cells.

PCR primers were designed for the selected 5 genes and validated by Real Time PCR. Two internal controls *18S* (Figure 5A–D) as well as *GAPDH* (Figure 5E–H) were used along with *ABCG2*, which acted as a control for SP phenotype. All 4 cell lines (A549, H1650, H460 and H1975) showed significant correlation with that of the microarray data for the 5 genes.

### PCA analysis

The 89 upregulated and 62 down regulated genes were used for Principle Component Analysis (PCA). The NCI Director's Challenge Set was derived using the Affymetrix Human Array 133A whereas our microarray was performed on Affymetrix Human Array Plus2.0 and hence we were able to match only 64 genes across the platform;these 64 genes were subjected to PCA. The PCA was performed to find the first principle component along which the samples show the largest variation. Using the Evince 2.5.5 software PCA was computed for the 64 genes and the risk scores for validation were tabulated for all the 64 genes. Through this PCA analysis we first established a 64 gene signature for the 4 NSCLC cell lines.

### Prognosis prediction

**Figure 6:**
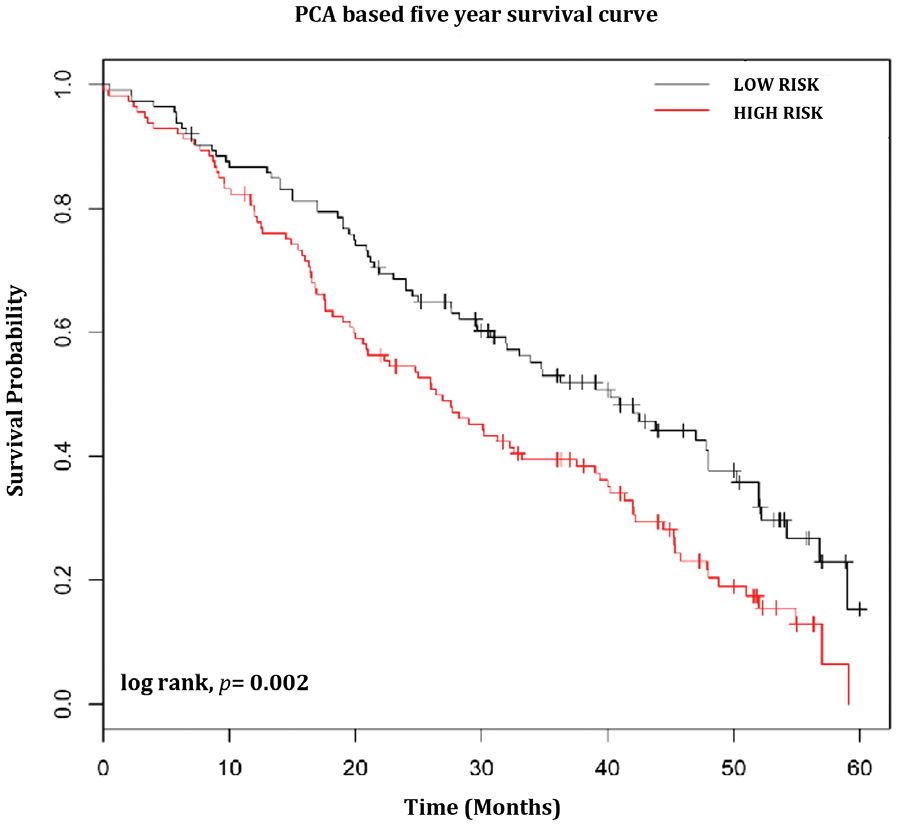
Principle Component Analysis (PCA) for 64 genes signature. The 89 up and 62 down regulated genes were used for PCA analysis. PCA was computed for the 64 genes and the risk scores for validation were tabulated for all the 64 genes. Through this PCA analysis we first established a 64 gene signature for the 4 NSCLC cell lines. The Kaplan-Meier analysis for the 64 genes signatures shows significantly poor prognosis for the differentially regulated genes.

A subset (n = 360) of the larger lung adenocarcinoma dataset was used for the study. The probe set IDs of the 89 up and 62 down regulated genes obtained using Human Genome U133A Plus 2.0 platform was compared with the probe set IDs of the NCI's dataset (used Human Genome U133A platform). This resulted in a total of 64 genes that matched with the other platform. Through the PCA analysis the risk score was dichotomized at the optimal cutoff and the 64 gene signature classified into low and high risk groups, respectively with significant difference in overall survival (*p* = 0.0002, Figure 6).

**Figure 7:**
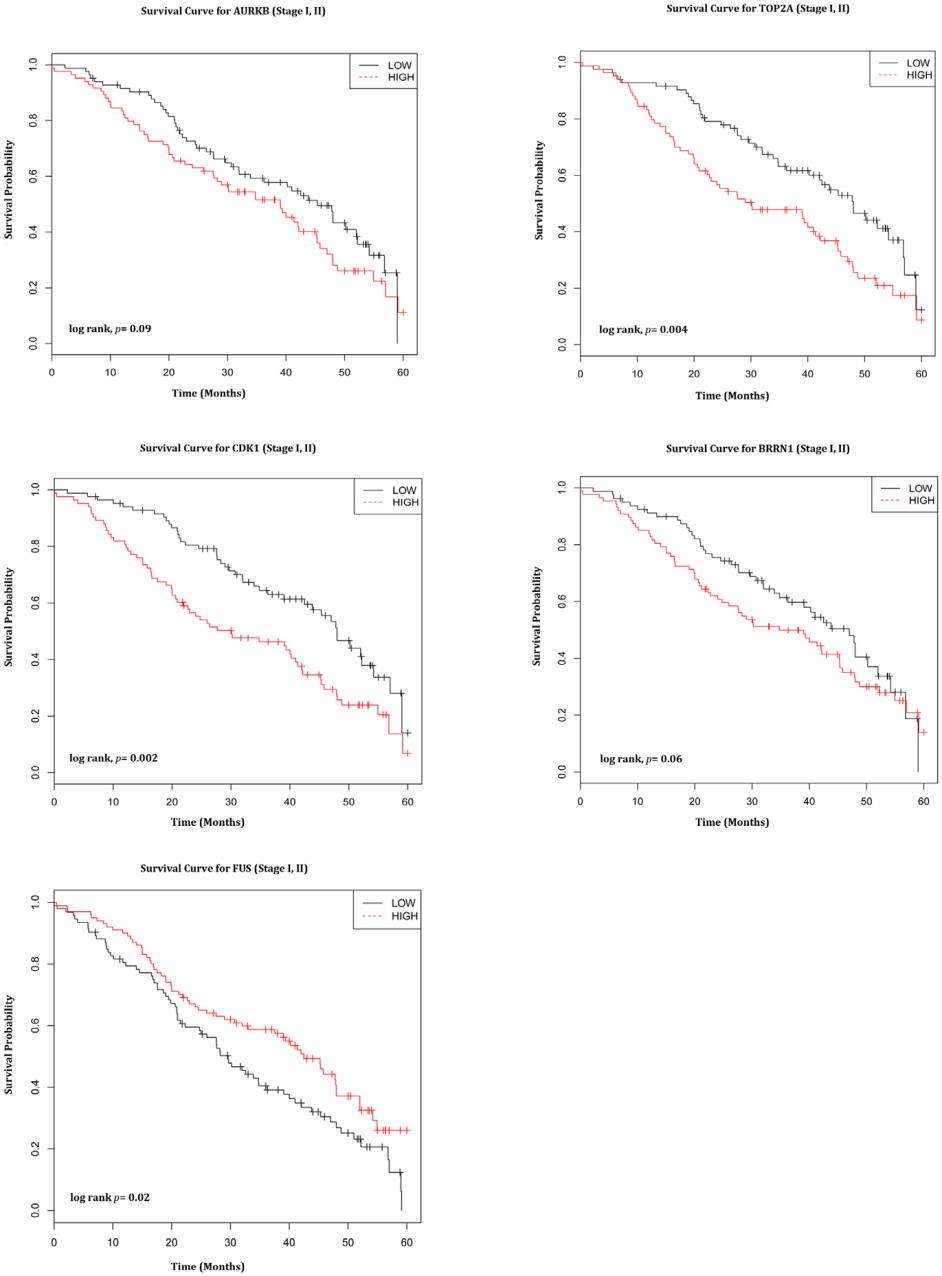
Kaplan-Meier Survival Curves for stage I, II patients from the NCI's Directors Challenge Set. Kaplan-Meier analysis showed a significant trend for 5 genes *AURKB*, *TOP2A*, *CDK1*, *BRRN1* and *FUS*. All these genes show poor survival in stage I, II patients in the NCI Director's challenge set.

The 5 genes from the most significant pathways were then used for independent survival prediction. Median expression values were used to dichotomize into low and high expression levels for 5 genes. Kaplan-Meier analysis of overall survival showed a significant trend for 5 genes namely *AURKB*, *TOP2A*, *CDK1*, *BRRN1* and *FUS* in 360 NCI Director's challenge set (Figure 7). The survival prediction that includes only stage I and II patients distinguished *AURKB*, *TOP2A*, *CDK1*, *BRRN1* and *FUS* at significance *p* values of 0.09, 0.004, 0.002,0.06 and 0.02 by log-rank test. Patients with lower expression levels of *AURKB*, *TOP2A*, *CDK1* and *BRRN1* had a significantly better prognosis than those patients with higher expression levels of these genes. The other gene, *FUS*, which is under expressed in SP cells (over expressed in MP cells) shows better survival of patients with high expression levels. The overall survival curve for these 5 genes that includes all stages also showed significant prognosis (Figure S1).

**Figure 8:**
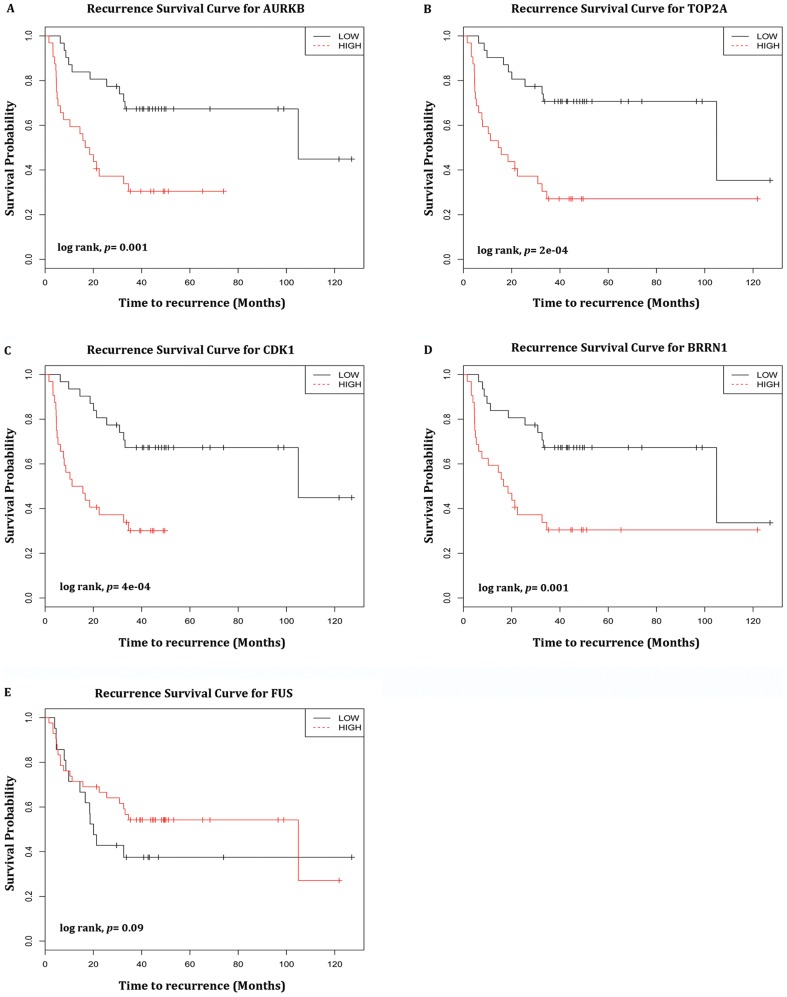
Recurrence Free survival curve for SKKU dataset. Kaplan-Meier analysis showed a significant trend for 5 genes *AURKB*, *TOP2A*, *CDK1*, *BRRN1* and *FUS*. All these genes show poor survival of patients in 63 adenocarcinoma samples from SKKU University.

Kaplan-Meier analysis was also carried out for 63 adenocarcinoma samples from SKKU (Figure 8) to estimate the survival probability following surgery. Results show lower expression levels of *AURKB*, *TOP2A*, *CDK*, *BRRN1* and higher expression levels of *FUS* are strongly associated with the 5-year survival probabilities. The high and low expression levels of *AURKB*, *TOP2A*, *CDK1*, *BRRN1* and *FUS* differ significantly indicated by their *p* values of 0.001, 2E-04, 4E-04, 0.001 and 0.09. This prognosis indicator shows patients with a high probability of tumor recurrence tend to be more likely to have treatment failure after surgery. This indicates that the high-risk probability shown by the survival curves is a good prognostic factor for lung cancer survival. These results suggest that a 5 gene signature from SP and MP cells can be used to predict prognosis of NSCLC patients.

## Discussion

Identifying gene expression signatures that capture altered key pathways in carcinogenesis may lead to the discovery of molecular subclasses and predict patient outcomes [26], [27]. Gene signatures provide a glimpse into critical molecular pathways, as they essentially serve as a bridge between clinical phenotypes and genomics. Indeed, the vast majority of biomarkers are functionally and biologically understood, in stark contrast with gene signatures. Moreover, biomarkers tend to be single-pathway-specific, whereas gene signatures may span multiple mechanisms [28].

The cancer stem cell hypothesis has gained significant traction over the past several years. An important criteria is that tumors with high percentages of cancer stem cells will be more aggressive, presumably because cancer stem cells are resistant to therapy [29]. Gene-expression profiling using microarrays or qRT-PCR has been shown to estimate the prognosis for patients with lung cancer [30]. Our selection of genes in the microarray dataset was validated in RT-PCR, and the patterns of gene expression found on microarray analysis correlated with that of RT-PCR. The results of RT-PCR performed on the 5 selected genes have been shown to correlate with the outcomes of lung adenocarcinoma.

We also tried to assess if any of the genes from the pathway analysis had Epithelial to Mesenchymal Transition (EMT) properties so that they can be considered as EMT signatures. Epithelial to mesenchymal transition (EMT) is a vital process for morphogenesis during embryonic development, but more recently it has also been implicated in the conversion of early stage tumors into invasive malignancies [31]. Progression of most carcinomas toward malignancy is associated with the loss of epithelial differentiation and by switching toward a mesenchymal phenotype, which is accompanied by increased cell motility and invasion. Recent studies have demonstrated that EMT plays a critical role not only in tumor metastasis but also in tumor recurrence that is believed to be tightly linked with the biology of cancer stem-like cells or cancer-initiating cells [32]. Evidence suggests that the acquisition of EMT is strongly associated with cancer cell invasion and tumor metastasis. Also studies have shown that cells with EMT phenotype share characteristics that are consistent with the signatures of cancer stem-like cells, which are associated with tumor recurrence and drug resistant phenotype and contribute to the demise of patients diagnosed with cancers [33]. For this analysis, we selected the top 10 significant pathways from the analysis and looked for genes that had cell adhesion properties. Only two EMT related genes *CD44* and *β-catenin* were involved in the pathways and these were used for survival prediction in the NCI director's challenge set (Figure S2). Both the genes showed no significance in the survival curve thus differentiating this 5 gene signature from the EMT property.

Recent studies have reported that the genes identified here are differentially expressed across multiple cancer types [34]. Differentially expressed genes with at least 2-fold changes between cancer and corresponding control tissues across seven cancer types were examined to find those genes common to multiple cancer types. This study showed a total of five genes amoung which two genes *CDK1* and *TOP2A* differentially expressed across five cancer types. The differences in the gene expression across different cancer types may indicate either a general or specific relevance of the gene to the corresponding cancers, which has been partially confirmed by the functional analysis. *CDK1*, up-regulated in five of the seven cancers studied, has been reported to be related to colon, prostate and stomach cancer, in view of its role in regulating the cell cycle, e.g. entry from G1 to S. *TOP2A*, again up-regulated in five of the seven cancers, has been reported to be associated with gastric [35], breast [36] and ovarian cancer [37], consistent with its function in DNA strand regulation. Both *CDK1* and *TOP2A* genes have been considered as multi-type cancer markers by a previous meta-analysis of cancer microarray data [38]. *TOP2A* encodes a DNA topoisomerase II, an enzyme that controls the topologic state of DNA during transcription. *TOP2A* is localized to the centromeric heterochromatin throughout most of meiotic prophase and suggests a meiotic function for *TOP2A* in addition to its role in chromatin condensation. This gene is currently the target of several anticancer agents, and a variety of its mutations have been associated with the development of drug resistance [39], [40].

Another study has shown gene expression profiles strongly differentiated smokers from non-smokers in lung tumors and early stage tumor tissue from non-tumor tissue consistent with an important role in lung carcinogenesis induced by smoking [41]. This helped to explore the impact of the smoking signature on survival from lung cancer in smokers. Results show cell cycle genes differentiating current from never smokers in the early stage tumor tissue samples one of them being *CDK1* gene. Mortality risk in smokers for gene expression differentiates current from never smokers in lung tumor and non-tumor tissue samples with *TOP2A* gene being one of them. In addition a member of the Aurora kinase family *AURKA* (closely associated with *AURKB*) involved in tumor progression has been found to be over expressed in smoking-related tumors [42]. The Aurora kinases are a conserved family of serine/threonine kinases that function in mitosis and meiosis. In human cell lines, *AURKB* functions in chromosome condensation, alignment, and segregation, as well as cytokinesis. In somatic cells, *AURKB* has been found at the midbody of anaphase cells and at the post-mitotic bridge of telophase cells, participating in chromatin modification, microtubule- kinetochore attachment, spindle checkpoint and cytokinesis [43]. Aurora kinases are over-expressed in a variety of tumor cell lines, suggesting that these kinases might play a role in tumorigenesis, and have already become potential targets for cancer diagnosis and therapy [44].

NK2-related homeobox transcription factor Nkx2-1 (also called Ttf-1 or Titf1) has been identified as a candidate suppressor of malignant progression in lung adenocarcinoma [45]. Data specifically link Nkx2-1 downregulation to loss of differentiation, enhanced tumor seeding ability and increased metastatic proclivity. Significant gene expression alterations distinguished T_nonMet_ (tumor non metastatic) from T_Met_ (tumor metastatic). A gene expression signature generated by comparing T_nonMet_ to T_Met/Met_ samples predicted patient outcome in human lung adenocarcinoma gene expression data sets [20], [46]. Our 5 gene signature consisting of *TOP2A*, *AURKB*, *BRRN1*, *CDK1* and *FUS* were all found in the Nkx2-1 corresponding gene signature. This shows an important significance since in human lung adenocarcinoma the expression of Nkx2-1 correlated with a mouse T_nonMet_ gene expression signature. The T_nonMet_ signature anti-correlated with an embryonic stem cell signature explaining that T_Met/Met_ cells have transitioned to a less differentiated and more stem-like state.

The 5 gene signature shown here is specific for lung adenocarcinoma. To strengthen this point we chose 75 squamous cell carcinoma data from the SKKU dataset and used for survival prediction. The analysis showed no significance for the squamous cell carcinoma data (Figure S3) thus highlighting our gene signature specific for lung adenocarcinoma. Our study supports the contention that it is feasible to construct a gene signature from significant pathways to predict clinical outcomes. The identification of five genes that are closely associated with the outcomes in patients with NSCLC could have clinical implications since this 5 gene signature could be useful in stratifying patients according to risk in treatment of the disease.

## Methods

### Side Population analysis

Four adenocarcinoma cell lines A549, H1650, H460 and H1975 were subjected to FACS (Fluorescence activated cell sorting) analysis for sorting SP and MP cells. The cell suspensions were labeled with Hoechst 33342 dye (Invitrogen) using the methods described by Goodell et al. [47] with modifications. Briefly, cells were resuspended at 1×10^6^/mL in prewarmed DMEM (Invitrogen-Life Technologies) with 2% FBS (Invitrogen-Life Technologies) and 10 mmol/L HEPES buffer (Invitrogen- Life Technologies). Hoechst 33342 dye was added at a final concentration of 5 mg/mL and the cells were incubated at 37°C for 90 min with intermittent shaking. At the end of the incubation, the cells were washed with ice-cold HBSS (Invitrogen-Life Technologies) with 2% FBS and 10 mmol/L HEPES, centrifuged down at 4°C, and resuspended in ice-cold HBSS containing 2% FBS and 10 mmol/L HEPES. Propidium iodide (Molecular Probes- Invitrogen) at a final concentration of 2 mg/mL was added to the cells to gate viable cells. Analyses and sorting were done on a FACSVantage SE (Becton Dickinson). The Hoechst 33342 dye was excited at 357 nm and its fluorescence was dual-wavelength analyzed (blue, 402–446 nm; red, 650–670 nm).

### Microarray and functional pathway analyses

Our present study focuses on lung adenocarcinoma and hence we chose 4 cell lines A549, H1650, H460 and H1975 that represented them. We used two cell lines that harbored mutations in K-Ras and two that had mutated EGFR. Since these are the most widely mutated genes in NSCLC, we wanted to focus on genes that were altered across the spectrum, irrespective of the upstream mutation. Two samples (SP, MP) each for 4 cell lines, so a total of 8 samples were used for microarray analysis. Total RNA extracted from SP (side population) and MP (main population) samples were used to generate cRNA targets, which were subsequently hybridized to Human Genome U133A plus 2.0 oligonucleotide probe arrays (Affymetrix, Santa Clara, CA) according to standard protocols. Raw data was processed by log_2_ transformation of the expression values, and the mean center expression level for each gene was determined. The data discussed in this publication have been deposited in NCBI's Gene Expression Omnibus through GEO Series accession number GSE36821. In brief, we identified genes that were over- or under-expressed in SP and MP from all 4 cell lines, whose expression was altered at least two fold. Further pathway analysis was carried out using MetaCore™ of GeneGo, Inc. MetaCore analyzes experimental high-throughput data in the context of pathways and networks that are ideal for data mining. It is a database of known molecular interactions, pathways and processes manually curated from published data and allow the user to visualize known biological systems within their data [48], [49], [50]. It also includes human protein–protein interactions, signal transduction, and metabolic pathways, and a variety of cellular functions and processes for signaling pathway analysis. This pathway analysis tool was used to obtain curated molecular interactions related to the differentially regulated genes.

### Real-time PCR validation

Real-time RT-PCR on SP and MP from the 4 NSCLC cell lines was used to confirm the expression levels of the identified signature genes in microarray platform. The number of cycles required to reach threshold fluorescence (Ct) and ΔCT for each sample relative to the control gene defines the expression pattern for a gene. The gene expression data were further analyzed using the 2^ΔΔCT^ method [51].

### Analysis of publicly available Microarray datasets

Gene expressions profiles analyzed in this study include 22,283 probes quantified with Affymetrix HG-U133A on 360 lung adenocarcinoma samples from Shedden et al., [20] and 63 adenocarcinoma samples from SKKU dataset [21]. The Harvard data from the NCI Director's challenge set was an outlier for our analysis and hence we removed 82 samples from the total 442 samples. Raw signal intensities for each probe set as they are contained in the *CEL* files were analyzed using the software package Bioconductor [52] (http://bioconductor.org). Expression values were normalized using MAS5.0 in R. Using mRNA expression profiles of the identified genes as predictors, a prognostic model can be constructed to stratify patients into low-risk and high-risk groups.

### PCA based gene signature

Principle component analysis (PCA) is a mathematical algorithm that reduces the dimensionality of the data while retaining most of the variation in the dataset [53]. By using few components, each sample can be represented by relatively few numbers instead of by values for thousands of variables. PCA was used for gene expression data for dimensionality reduction and removing possible collinear expression of genes. Risk scores were calculated for the differentially regulated genes using the Evince 2.5.5 of UmBio. A patient's risk score was calculated as the sum of the levels of expression of each gene, as measured by microarray analysis, multiplied by the corresponding regression coefficients [54]. Patients were classified as having a high-risk gene signature or a low-risk gene signature, with the 50th percentile (median) of the risk score as the threshold value.

### Statistical analysis

Statistical analyses were done using R package [55] (http://www.r-project.org/). To determine whether the gene signature correlates with poor prognosis, we performed Kaplan-Meier and log-rank test (for *p* value) analysis of overall survival. Overall survival time was calculated from the date of surgery until death or the last follow-up contact. Recurrence-free survival time was defined as the time interval between the date of surgery and the date of disease recurrence or death from any cause, whichever came first, or date of last follow-up evaluation. The Kaplan–Meier method was used to estimate overall survival and relapse-free survival. Differences in survival between the high-risk group and the low-risk group were analyzed with the log-rank test. A *p* value of less than 0.05 was considered to indicate statistical significance, and all tests were two-tailed. All the analyses were performed with packages in R unless otherwise specified.

## Conclusion

The development of microarray methods for large-scale analysis of gene expression makes it possible to search systematically for gene signatures of cancer classification and outcome prediction in a variety of tumor types. The 5 gene signature highlights effective identification of lung adenocarcinoma patients with poor prognosis. Cancer biologists and clinical researchers could focus attention on the relatively small number of genes identified here showing differential gene expression patterns. Our studies show that gene expression profile from a tumor initiating side-population cell may represent both a useful predictor of treatment response and potentially a target for effective treatment.

## Supporting Information

Figure S1 **Overall Survival Curves for the NCI's Directors Challenge Set.** Kaplan-Meier analysis showed a significant trend for 5 genes *AURKB*, *TOP2A*, *CDK1*, *BRRN1* and *FUS*. All these genes show poor survival of patients in 360 NCI Director's challenge set.
(TIF)

Figure S2 **Survival Curves for the EMT related genes in NCI's Directors Challenge Set.** We selected the top 10 significant pathways from our analysis and assessed for genes that had cell adhesion properties. Only two EMT related genes CD44 and beta-catenin were involved in the pathways and these were used for survival prediction in the NCI director's challenge set. Both the genes in Kaplan-Meier analysis showed no significance in the survival thus differentiating our 5 gene signature from the EMT property.
(TIF)

Figure S3 **Recurrence Free survival curve for SKKU Squamous Cell Carcinoma dataset.** Squamous cell carcinoma data (n = 75) from the SKKU (Sungkyunkwan University) dataset was used for survival prediction for the 5 genes. Previously we used adenocarcinoma data (n = 63, Figure 8) from the same dataset and predicted prognostic significance. The analysis here showed no significance for the squamous cell carcinoma data thus highlighting our gene signature specific for lung adenocarcinoma.
(TIF)
