# Supplementary figures and images for: A Novel Five Gene Signature Derived from Stem-Like Side Population Cells Predicts Overall and Recurrence-Free Survival in NSCLC
Source: PLoS One. 2012 Aug 29;7(8):e43589. doi: 10.1371/journal.pone.0043589 (PMC3430700; doi:10.1371/journal.pone.0043589)

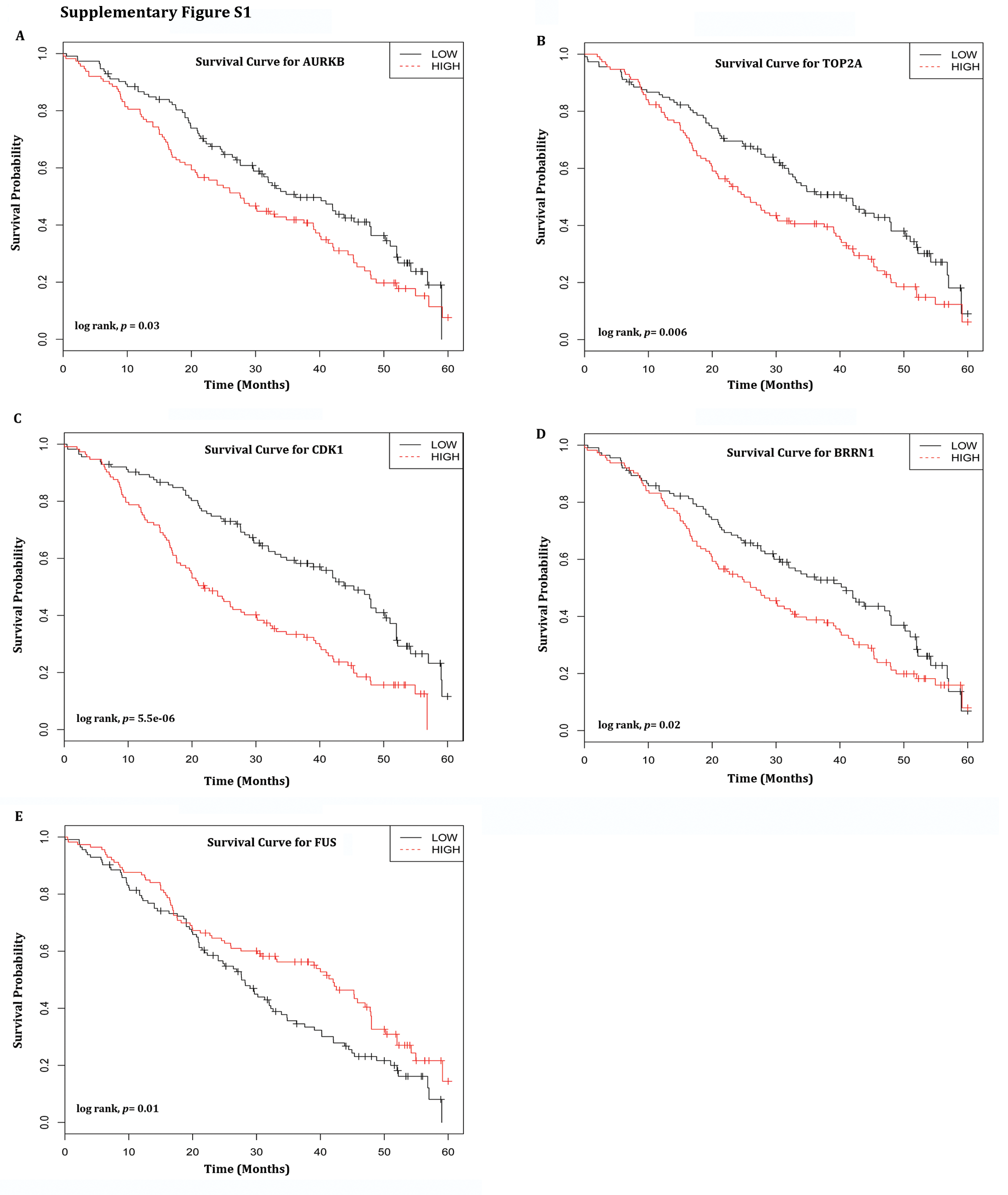

Supplement: Figure S1 — Overall Survival Curves for the NCI's Directors Challenge Set. Kaplan-Meier analysis showed a significant trend for 5 genes AURKB, TOP2A, CDK1, BRRN1 and FUS. All these genes show poor survival of patients in 360 NCI Director's challenge set. (TIF) [file pone.0043589.s001.tif]

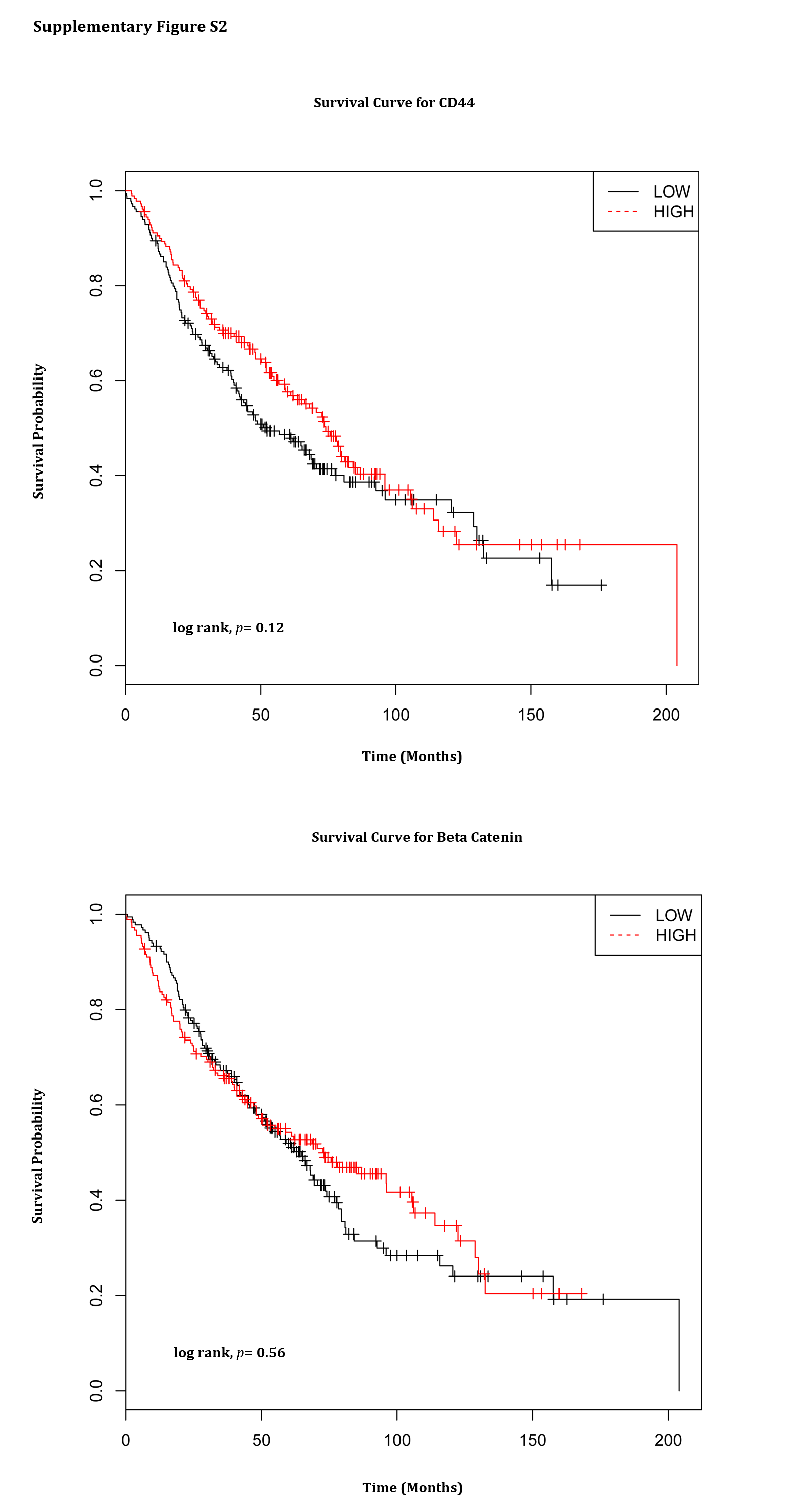

Supplement: Figure S2 — Survival Curves for the EMT related genes in NCI's Directors Challenge Set. We selected the top 10 significant pathways from our analysis and assessed for genes that had cell adhesion properties. Only two EMT related genes CD44 and beta-catenin were involved in the pathways and these were used for survival prediction in the NCI director's challenge set. Both the genes in Kaplan-Meier analysis showed no significance in the survival thus differentiating our 5 gene signature from the EMT property. (TIF) [file pone.0043589.s002.tif]

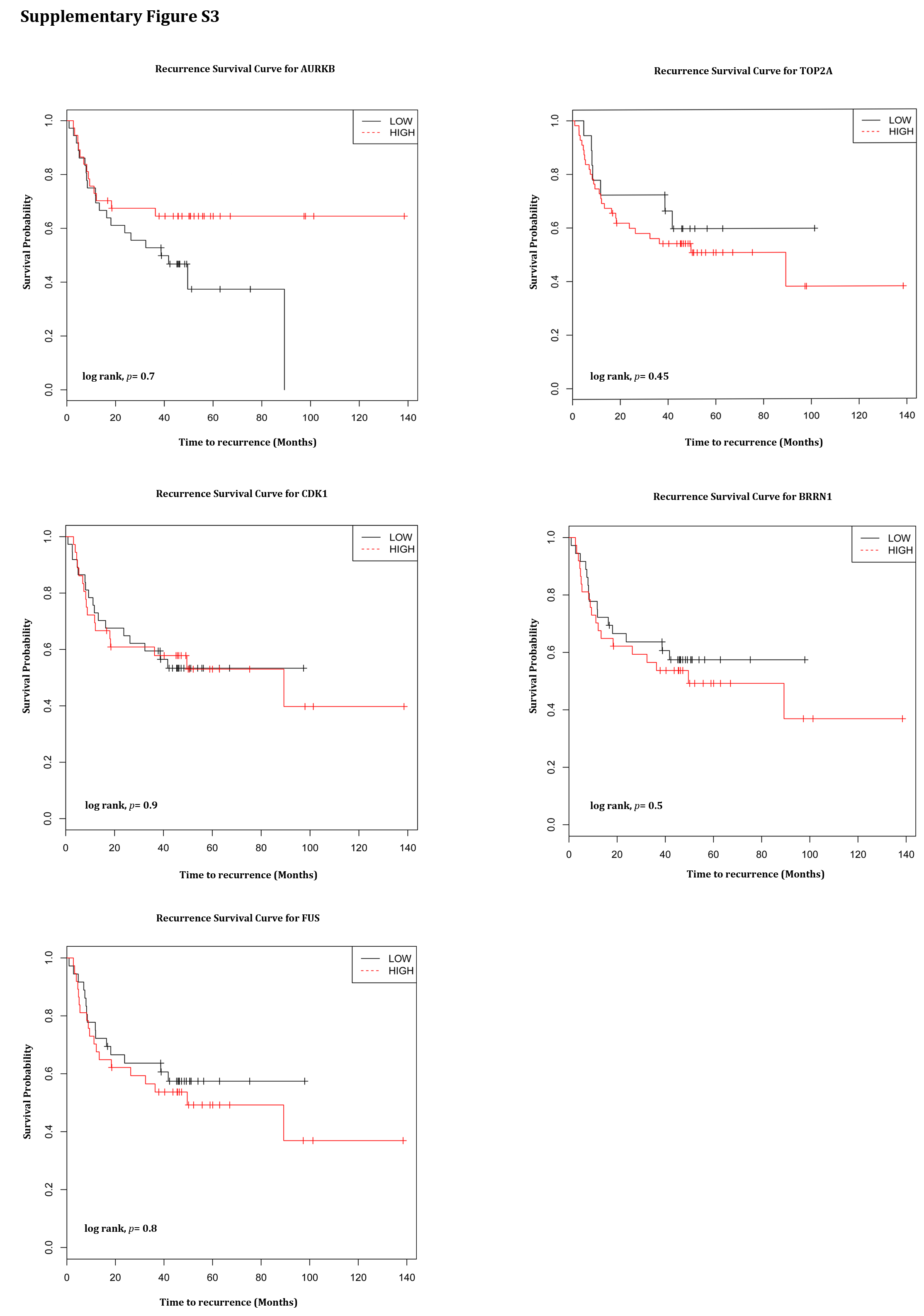

Supplement: Figure S3 — Recurrence Free survival curve for SKKU Squamous Cell Carcinoma dataset. Squamous cell carcinoma data (n = 75) from the SKKU (Sungkyunkwan University) dataset was used for survival prediction for the 5 genes. Previously we used adenocarcinoma data (n = 63, Figure 8) from the same dataset and predicted prognostic significance. The analysis here showed no significance for the squamous cell carcinoma data thus highlighting our gene signature specific for lung adenocarcinoma. (TIF) [file pone.0043589.s003.tif]
